# A Novel Ag/AgCl/Ag_3_PO_4_ Nanocomposite Demonstrates Potent Antibacterial Activity Against Multidrug-Resistant and Pan-Resistant Pathogenic Isolates of *Acinetobacter baumannii*

**DOI:** 10.3390/microorganisms14081682

**Published:** 2026-07-31

**Authors:** Victor Hugo Montini, Laura Santana Buso, Anastácia Nikolaos Deonas, Gabriel Henrique Maximino Santos, Bruna Carolina Gonçalves, Maria Luiza Francisconi Lubanco Thomé, Paulo Rogério Catarini da Silva, Diego Prudencio dos Santos, Cesar Ricardo Teixeira Tarley, Admilton Gonçalves de Oliveira, Danielle Lazarin Bidóia, Renata Katsuko Takayama Kobayashi, Gerson Nakazato

**Affiliations:** 1 Laboratory of Basic and Applied Bacteriology, State University of Londrina, P.O. Box 10.011, Londrina 86057-970, Brazil; 2 Laboratory of X-Ray Analysis, Multiuser Research Laboratory Center, State University of Londrina, P.O. Box 10.011, Londrina 86057-970, Brazil; 3 Department of Chemistry, State University of Londrina, P.O. Box 10.011, Londrina 86057-970, Brazil; 4 Laboratory of Microbial Biotechnology, State University of Londrina, P.O. Box 10.011, Londrina 86057-970, Brazil; 5 Department of Clinical Analysis and Biomedicine, State University of Maringá, P.O. Box 10.011, Maringá 87020-900, Brazil

**Keywords:** antimicrobial, Gram-negative, HAI, nanosilver, nanobiotechnology

## Abstract

*Acinetobacter baumannii* is a microorganism of major global clinical importance, whose carbapenem-resistant phenotype is considered a priority target for the development of new antimicrobial agents. Carbapenem-resistant *A. baumannii* is associated with healthcare-associated infections and exhibits high mortality rates, prolonged hospital stays, and extensive use of intensive care units. As a control strategy, green-synthesized silver nanoparticles have gained increasing attention. Based on this, the aim of this study was to synthesize a novel triphasic silver nanocomposite, characterize it, and evaluate its antibacterial activity against extensively drug-resistant and pan-drug-resistant isolates. The nanocomposite was synthesized using cell-free bacterial supernatant and was physicochemically characterized regarding concentration, size, morphology, and crystallinity. Its antibacterial activity was assessed using broth microdilution and time–kill assays, followed by toxicity assays using human erythrocytes. A nanoscale and stable nanocomposite was obtained and was composed of metallic silver, silver chloride, and silver phosphate, which exhibited antibacterial activity against all tested isolates. In the toxicity assay, the nanocomposite demonstrated low toxicity and a high selectivity index. These findings demonstrate that Ag/AgCl/Ag_3_PO_4_ nanocomposite exhibits strong activity against resistant phenotypes for which therapeutic options are limited, highlighting its potential as a control strategy against this pathogen.

## 1. Introduction

Antimicrobial resistance and the increasing incidence of multidrug-resistant pathogens are classified as a global public health problem, with projected impacts of up to 10 million deaths annually and a reduction in the global gross domestic product (GDP) of up to 3.8% from 2050 onward [1,2].

It is estimated that, in 2019, approximately 4.95 million deaths were associated with infections caused by multidrug-resistant (MDR) bacterial pathogens, and 1.27 million were directly attributable to MDR infections worldwide. In Latin America, 338,000 deaths were associated with MDR, and 84,300 were attributable to MDR [1,3]. The most affected super-regions, with high mortality rates across all age groups, include Latin America itself [4].

Among the main bacterial pathogens associated with these deaths is *A. baumannii*, responsible for 45,300 deaths in the Americas, of which 14,300 were directly attributable to *A. baumannii* resistant to at least one antimicrobial agent. Of the deaths caused by resistant *A. baumannii*, 6160 were due to carbapenem-resistant *A. baumannii* (CRAB) and 4560 to fluoroquinolone-resistant *A. baumannii* [3].

*A. baumannii* is a Gram-negative opportunistic pathogen and an etiological agent of respiratory infections such as ventilator-associated pneumonia (VAP), bloodstream infections, skin and soft tissue infections, especially in severe burns and wounds, urinary tract infections, and meningitis. It is one of the main pathogens responsible for healthcare-associated infections (HAIs) [5]. Its association with HAIs, high mortality rate, increasing resistance trends, and limited treatment options led the World Health Organization (WHO) to classify CRAB as a critical priority pathogen for research and development of new antibiotics [6].

Infections caused by CRAB tend to have higher mortality rates than infections caused by susceptible strains, as well as longer hospital stays, higher rates of intensive care unit utilization, and increased readmission rates [7]. In fact, higher mortality rates have been reported among patients with CRAB infections than among those with carbapenem-susceptible *A. baumannii* infections involving the bloodstream, urinary tract, wounds, and other infection sites.

As an alternative for the treatment of *A. baumannii* and CRAB, the use of silver nanoparticles has been highlighted, particularly due to their broad-spectrum activity, with reports in the literature demonstrating effective action against *A. baumannii* [8].

As a therapeutic possibility, silver nanoparticles (AgNPs) have been proposed, especially due to their broad-spectrum action and rapid kinetics. Their antibacterial activity is mainly associated with cellular redox imbalance and the generation of oxidative stress, which can disrupt the bacterial cell wall and cell membrane, inhibit electron transport, alter essential cellular functions, and ultimately cause damage to genetic material [8].

Nanoparticles are nanostructures ranging from 1 to 100 nm and may consist of a metallic core, as in the case of metallic nanoparticles. These metallic nanoparticles can be produced through green synthesis, which has attracted considerable attention because it employs biological resources such as microorganisms (e.g., bacteria and fungi) and plants [8]. However, undesirable by-products may form during the synthesis of Ag^0^ nanoparticles, such as silver chloride and silver phosphate. These compounds are rarely investigated as antibacterial agents, resulting in a limited understanding of their individual and combined antimicrobial properties.

Based on this, the present study proposes to explore the synthesis and physicochemical and biological characterization of these species, as well as their antibacterial activity against extensively drug-resistant and pan-drug-resistant clinical isolates, in addition to evaluating their toxicity toward human cells.

## 2. Materials and Methods

### 2.1. Synthesis and Characterization

#### 2.1.1. Synthesis of Nanocomposite

The nanocomposite was synthesized according to document BR 10 2024 013609 8 [9]. Briefly, a silver nitrate solution was mixed with a solution containing cell-free supernatant (CFS) of *Escherichia coli* obtained by submerged liquid fermentation. Subsequently, the solution was irradiated with ultraviolet C light using a germicidal UV lamp for 1 h at room temperature. In this methodology, the cell-free supernatant (CFS) plays a dual role in the synthesis process. Together with UV-C irradiation, it contributes to the reduction of silver ions present in the solution, promoting the formation of silver-containing nanoparticles. Additionally, biomolecules present in the CFS act as capping and stabilizing agents, coating the resulting nanocomposite and contributing to its colloidal stability.

After the synthesis period, the nanoparticles dispersion was frozen at −20 °C, followed by freezing at −80 °C, and then lyophilized using an Alpha 1–2 LD plus (Germany) lyophilizer for 48 h. The lyophilized material was weighed and subsequently resuspended in sterile ultrapure water at a final concentration of 1 mg/mL.

#### 2.1.2. Dynamic Light and Electrophoretic Light Scattering

The hydrodynamic diameter and polydispersion were calculated by dynamic light scattering (DLS) while the zeta potential was determined by electrophoretic light scattering (ELS) using a Zetasizer Pro Blue instrument (Malvern Panalytical, Malvern, UK).

#### 2.1.3. Transmission Electron Microscopy

The size and morphology of the dispersion were analyzed using transmission electron microscopy (TEM) with a JEM 1400 electron microscope (JEOL, Tokyo, Japan) at the State University of Maringá. For analysis, 10 µL of the nanoparticles dispersion was deposited onto resin-coated carbon grids and dried overnight at room temperature. The acquired images were analyzed using ImageJ 1.54p software.

#### 2.1.4. X-Ray Diffraction

X-ray diffraction (XRD) measurements were performed at the X-ray Diffraction Laboratory (Multiuser Laboratory of the Office of Research and Graduate Studies) at State University of Londrina, using a PANalytical X’Pert PRO MPD diffractometer (Malvern Panalytical, Malvern, UK) with CuKα radiation, employing the θ–2θ technique. The voltage and current used were 40 kV and 30 mA, respectively. The 2θ scanning range was from 5 to 80°, with an angular step of 0.0394°. The counting time per point was 97.92 s.

To minimize possible preferred orientation effects during sample preparation and to homogenize measurements across the sample surface, the samples were continuously rotated during acquisition with a period of 1 s. Instrumental broadening was corrected by measuring a silicon standard.

#### 2.1.5. Atomic Absorption Spectrometry

The concentration of ionic silver was quantified using flame atomic absorption spectrometry (FAAS) with an AA-7000 spectrometer (Shimadzu, Kyoto, Japan), equipped with a hollow cathode lamp operating at a current of 10 mA and a wavelength of 328.1 nm. The flame used consisted of an acetylene/air mixture, operating at flow rates of 2.4 L min^−1^ and 15.0 L min^−1^, respectively.

The concentration of elemental silver was determined by linear regression using standard silver solutions ranging from 0 to 10 mg/L of AgNO_3_ dissolved in ultrapure water.

#### 2.1.6. Fourier Transformated Infrared Spectroscopy

The transmittance of the CFS and the nanoparticles dispersion was measured using a Fourier transform infrared (FTIR) spectrophotometer (IR Prestige-21, Shimadzu) with KBr pellets, at a resolution of 4 cm^−1^ and 32 scans, over a wavenumber range of 400–4000 cm^−1^.

### 2.2. Antibacterial Assays

#### 2.2.1. Strain and Isolates

Seven *A. baumannii* resistant isolates kindly provided by the University Hospital of Londrina–PR and one commercial strain were used (Table 1).

#### 2.2.2. Broth Microdilution

The strain and isolates were subjected to a broth microdilution assay to determine the minimum inhibitory concentration (MIC) and minimum bactericidal concentration (MBC) of nanoparticles, following the methodology described in CLSI guidelines M07-A and M26 [10,11].

Briefly, a final inoculum of 10^6^ CFU/mL of A*. baumannii*, prepared in Mueller–Hinton broth, was mixed in 96-well polystyrene plates at a 1:1 ratio with nanoparticles previously diluted in Mueller–Hinton broth (0.26–16.48 μg/mL) in triplicate. After mixing, the plates were incubated overnight at 37 °C for MIC determination. The MIC was determined based on the presence or absence of visible bacterial growth.

For MBC determination, 10 µL from clear wells was subcultured in triplicate onto Mueller–Hinton agar plates. The plates were incubated overnight at 37 °C, and CFUs were counted. The MBC was determined as the lowest concentration capable of reducing the initial bacterial inoculum by 99.9%. The MBC:MIC ratio was used to determine whether the compound was bactericidal (≤4) or bacteriostatic (4–32) against the isolates [12].

#### 2.2.3. Time–Kill Assay

The kinetics of action of nanoparticles were evaluated using a time–kill assay following the methodology described by M26 [11]. Briefly, in 1 mL, an inoculum of 10^5^ CFU/mL in Mueller–Hinton broth was exposed to nanoparticles at the MBC for 0, 1, 2, 4, 6, 8, 12, and 24 h at 37 °C. After each time point, 10 µL was subcultured in triplicate onto Mueller–Hinton agar plates, incubated overnight at 37 °C, and CFUs were subsequently quantified to construct a growth and death kinetics curve using GraphPad 9 software.

Based on the resistance phenotype, the strain *A. baumannii* ATCC 19606 and the isolates *A. baumannii* Ab846 and *A. baumannii* Ab860 were selected for this assay. Curve data were used to calculate the area under the curve (AUC), also using GraphPad 9.

### 2.3. In Vitro Toxicity

#### Hemolysis Assay

The hemolysis assay was performed according to Scandorieiro et al. (2016), with approval from the Human Research Ethics Committee (CAAE 47661115.0.0000.5231, No. 1.268.019—UEL) (Document S1) [13]. Approximately 8 mL of human blood was collected in a heparinized tube. Subsequently, 1 mL was transferred to a sterile microtube and centrifuged at 5000 rpm for 5 min at 4 °C. The plasma was discarded, and 60 µL of erythrocyte pellet was collected and added to 940 µL of 0.1 M PBS.

A total of 100 µL of the resulting 6% suspension was then mixed at a 100 µL of PBS containing the nanoparticles in sextuplicate. The negative control consisted of PBS without nanoparticles, and the positive control consisted of PBS with 1% Triton X-100. The plates were incubated for 3 h at 37 °C, after which 100 µL of the supernatant was analyzed in a spectrophotometer at 570 nm to assess hemoglobin release.

The hemoglobin release rate (%) was calculated according to the following:

$$\left(\frac{OD\;treatment-OD\;negative\;control}{OD\;positive\;control-OD\;negative\;control}\right)*100$$

The cytotoxic concentration (CC_50_) was defined as the concentration required to induce 50% hemolysis of erythrocytes compared with the untreated control. The CC_50_ value was determined by linear regression analysis. The selectivity index (SI) was calculated as the ratio between CC_50_ and MIC (SI = CC_50_/MIC).

## 3. Results

**Figure 1:**
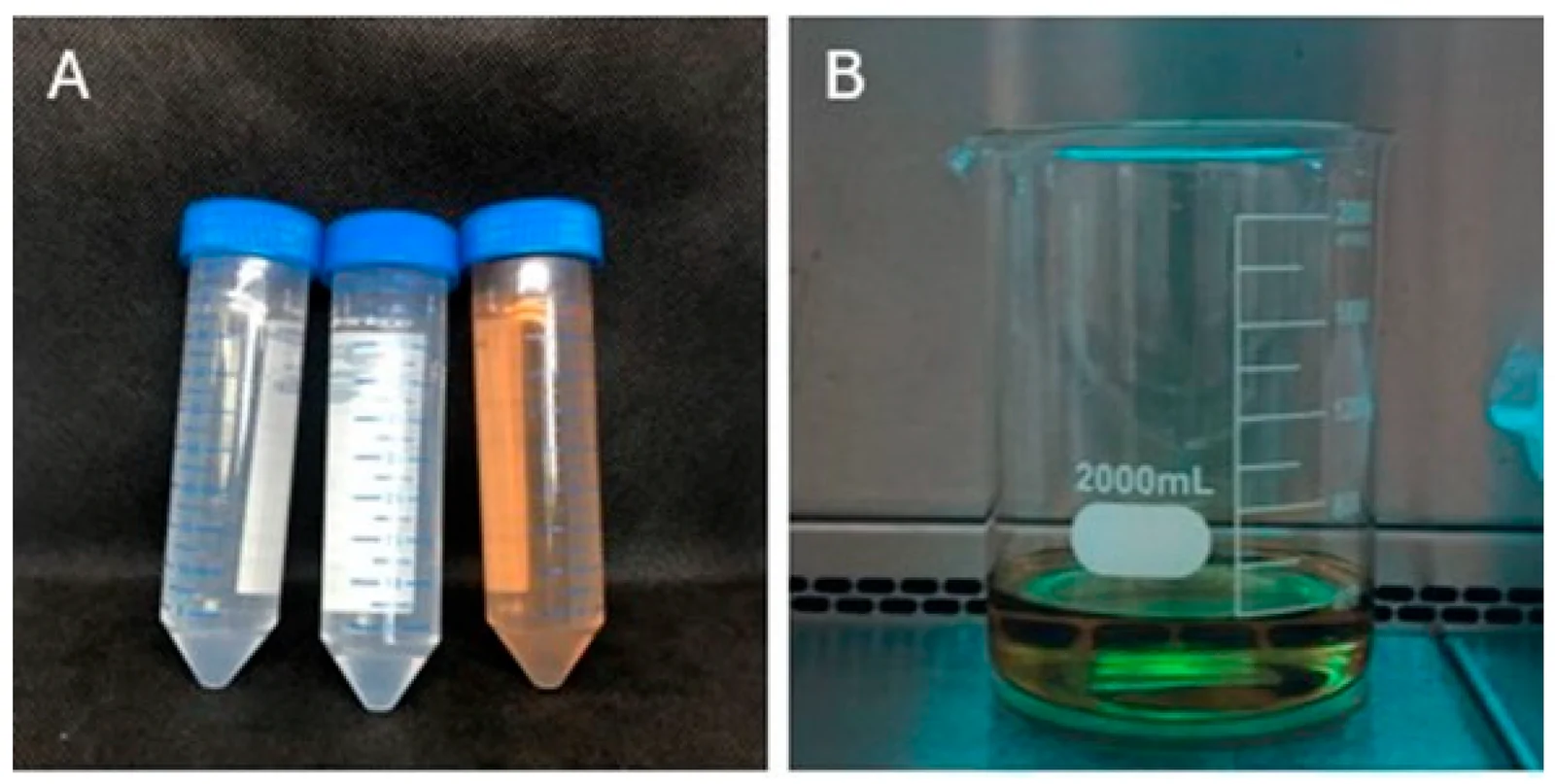
From left to right: ultrapure water, mixture of AgNO_3_ and cell-free supernatant prior to irradiation, and the mixture after 1 h of irradiation (**A**) and mixture after 1 h of irradiation (**B**).

After the standardized period, the solution containing the cell-free supernatant and AgNO_3_ showed a color change to light brown, indicating the formation of colloidal silver (Figure 1). This color change has been observed in studies using plant extracts, bacterial cells or metabolites, fungi, and chemical syntheses, indicating the formation of AgNPs [8,14,15,16]. The optical phenomenon responsible for this coloration is due to the absorption of blue light by AgNPs smaller than 40 nm and the combined emission of red and green wavelengths, resulting in a yellowish coloration [17].

**Figure 2:**
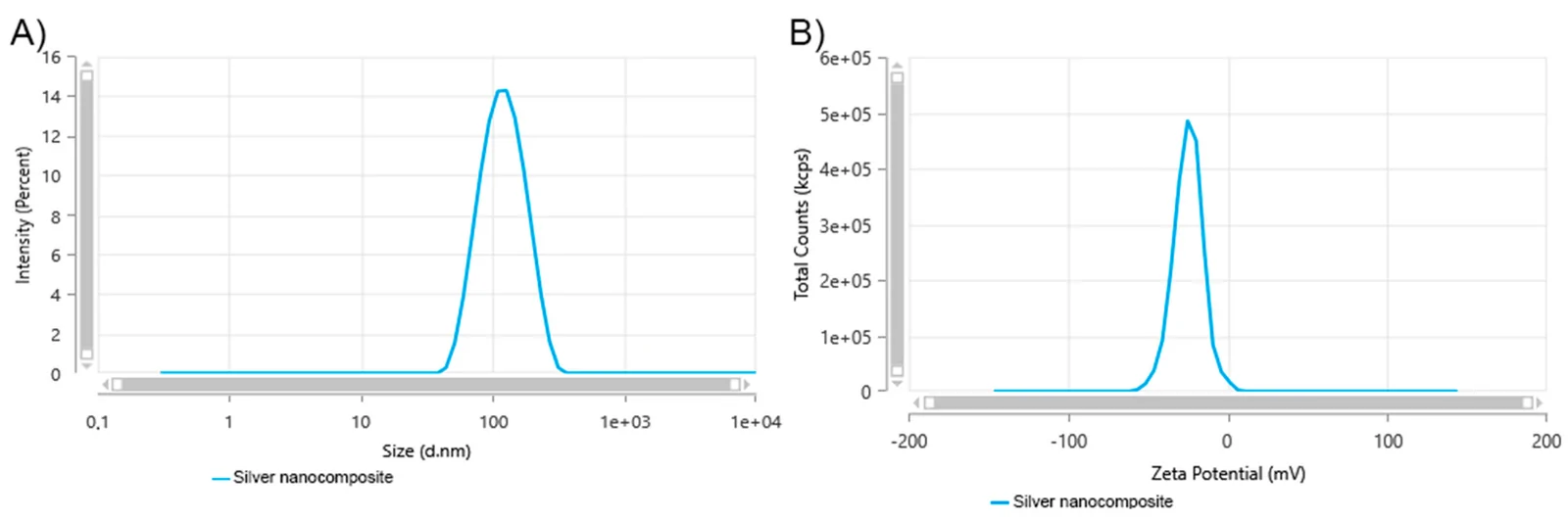
Size distribution by intensity (**A**) and zeta potential by total counts (**B**).

DLS and ELS analyses revealed that the nanocomposite presents an average hydrodynamic diameter of 101.754 ± 0.468 nm, a polydispersity index (PDI) of 0.205 ± 0.01 (Figure 2A), and a zeta potential of −24.686 ± 0.69 mV (Figure 2B). These results confirm its nanoscale nature and indicate colloidal stability.

According to widely used criteria in the literature, zeta potential values in the ranges of ±0–10 mV, ±10–20 mV, ±20–30 mV, and >±30 mV classify nanoparticles as very unstable, moderately unstable, stable, and highly stable, respectively [18]. Thus, the obtained value suggests that the nanocomposite exhibits satisfactory colloidal stability.

Regarding the polydispersity index, particles with PDI ≤ 0.1 are considered highly monodisperse; between 0.1–0.4, moderately polydisperse; and >0.4, highly polydisperse. Based on this classification, the nanocomposite can be characterized as moderately polydisperse.

**Figure 3:**
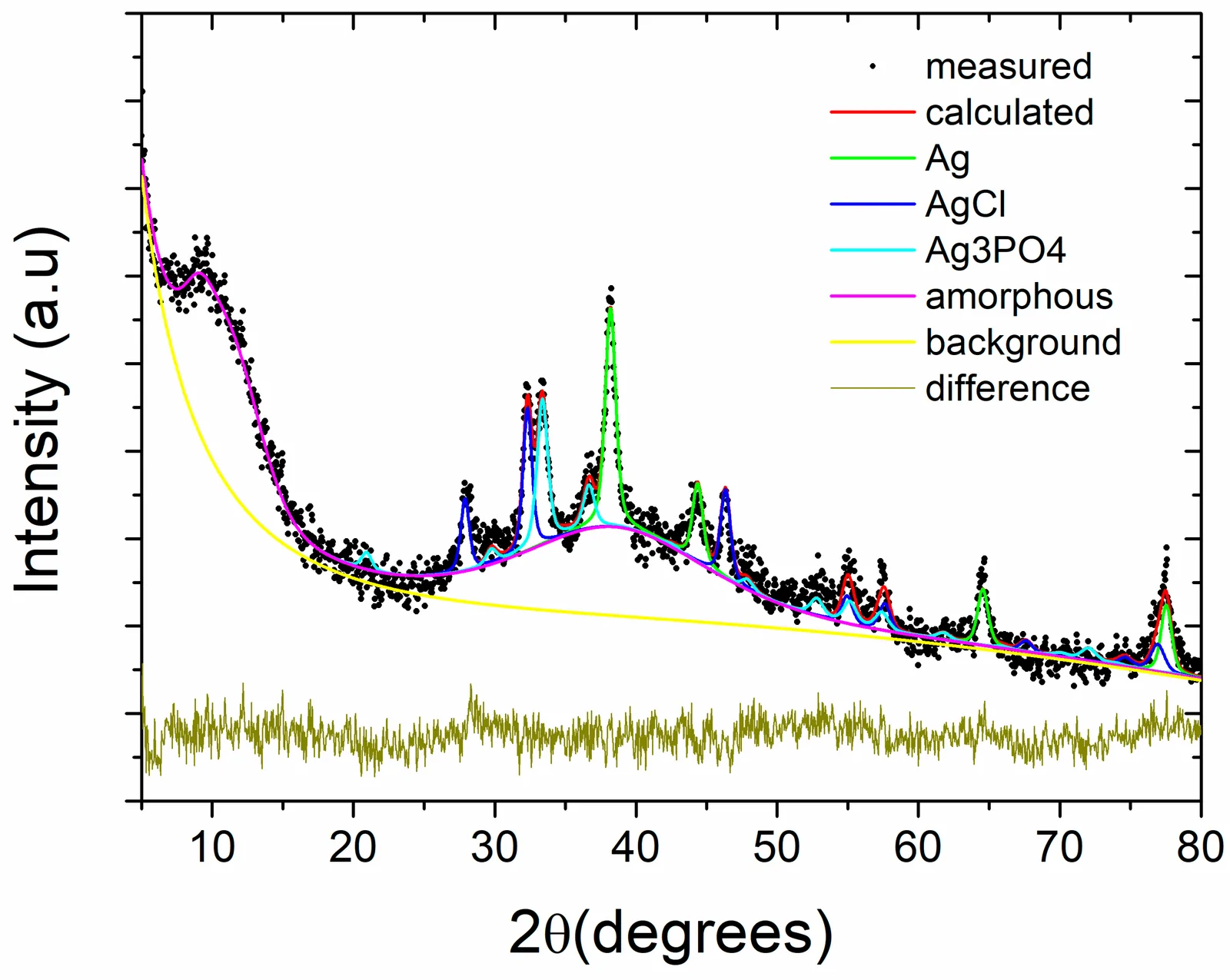
X-ray diffraction pattern of the lyophilized Ag/AgCl/Ag_3_PO_4_-NP suspension showing peaks corresponding to the different phases.

The X-ray diffraction pattern (Figure 3) revealed the presence of characteristic peaks corresponding to metallic Ag, AgCl, and Ag_3_PO_4_ phases, identified by comparison with the respective JCPDS cards (Table S3). These data confirm the multiphase composition of the Ag/AgCl/Ag_3_PO_4_ nanocomposite.

From the FWHM and peak position (Table S3), it was possible to determine the crystallite size and microstrain using the Scherrer, Williamson–Hall, and Size–Strain Plot equations (Table 2).

These data confirm the triphasic nature of the nanocomposite and the nanoscale crystallite size. The low microstrain values indicate that the broadening of the diffraction peaks is mainly associated with the reduced crystallite size rather than significant lattice strain.

**Figure 4:**
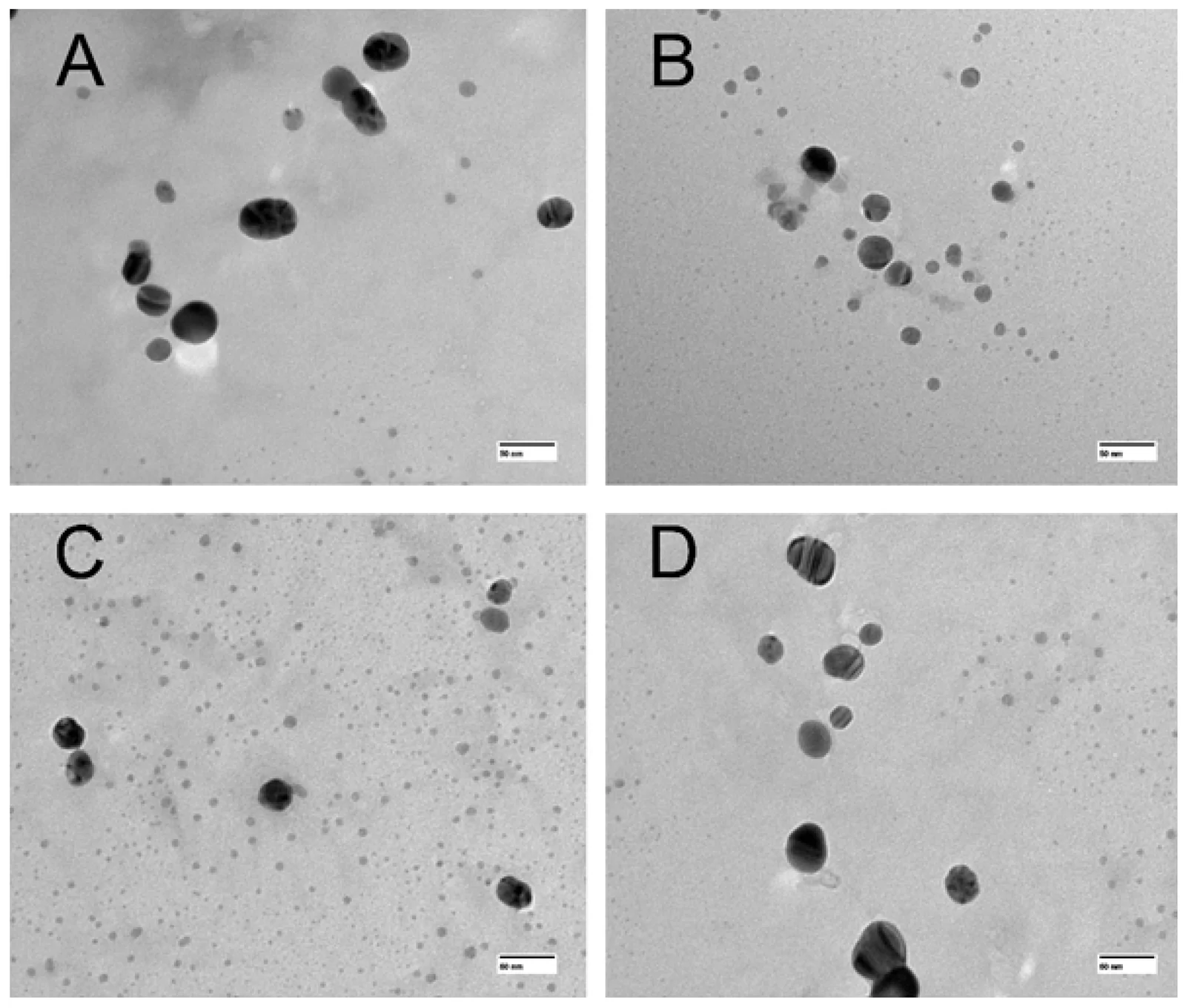
Transmission electron micrography of the nanoparticles (**A**–**D**). Scale bar = 50 nm.

The nanoparticle size was further analyzed by transmission electron microscopy (TEM) (Figure 4). Using ImageJ software, it was possible to determine the average size of the nanocomposite as 21.15 ± 12.52 nm, with an oval, slightly elongated, or spherical morphology. These values are consistent with those obtained through analytical modeling based on XRD data.

The difference between the sizes obtained by TEM and DLS is related to the fact that DLS measures the hydrodynamic diameter, accounting for the coating layer and assuming, according to its model, that the nanoparticles are perfectly spherical.

The quantification of elemental silver in the dispersion of 827 μg/mL corresponded to 82.7% of the total sample mass (Table S2; Figure S1). This value is higher than the theoretical silver content expected for the AgCl and Ag_3_PO_4_ phases, indicating a non-stoichiometric composition of the nanocomposite, with the presence of excess metallic silver (Ag^0^). This result, together with the dark coloration of the dispersion, supports the data obtained by X-ray diffraction, supporting the coexistence of the Ag/AgCl/Ag_3_PO_4_ phases.

**Figure 5:**
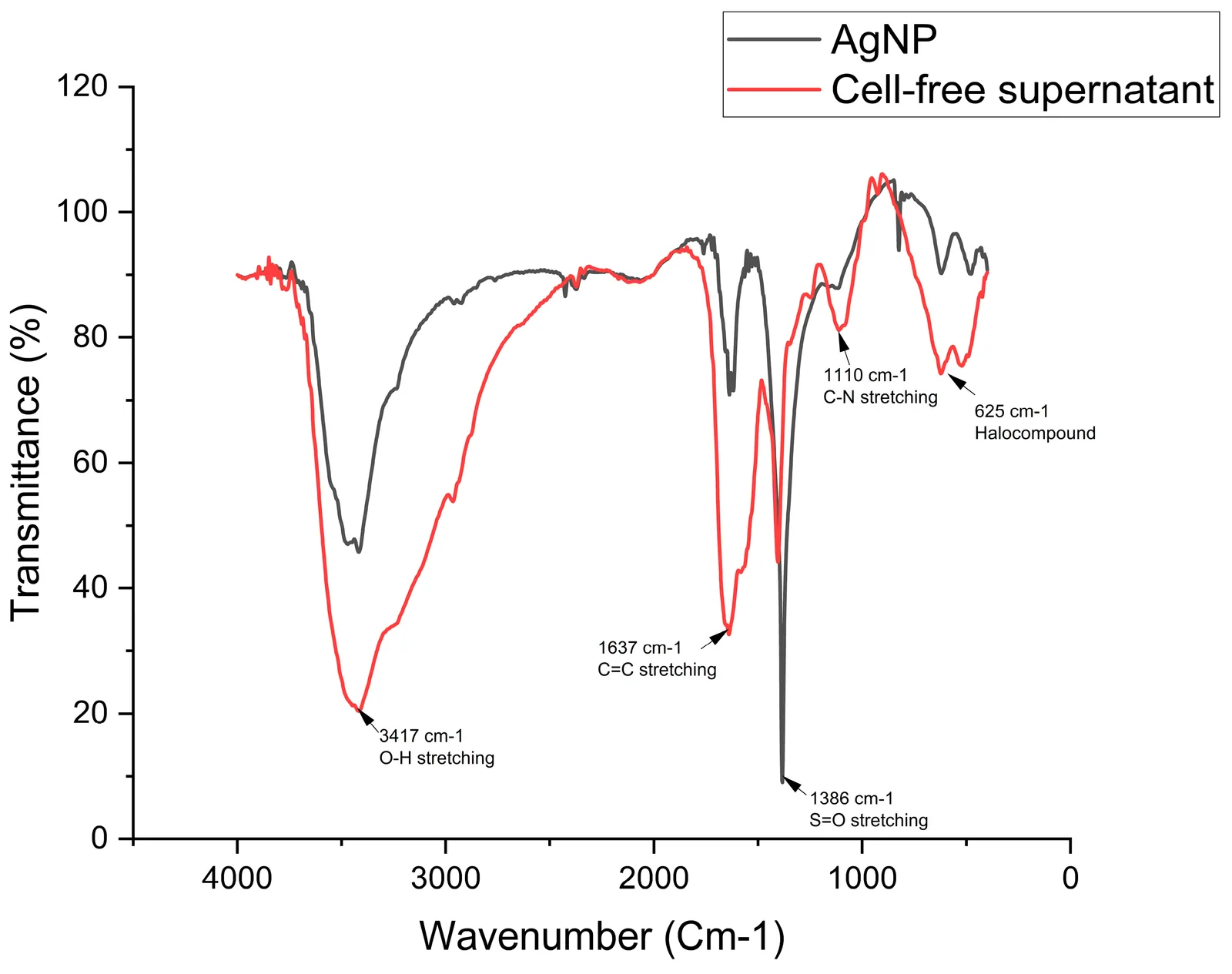
FT-IR spectrum of the cell-free supernatant and the silver nanoparticles obtained. Peaks at different wavenumbers are observed, indicating the presence of multiple organic functional groups.

FTIR analysis revealed peaks corresponding to various organic functional groups, demonstrating the presence of organic matter acting as a coating for the nanocomposite (Figure 5). Peaks at 3417 cm^−1^ correspond to O–H stretching vibrations of alcohols. Peaks at 1637 cm^−1^ correspond to C=C stretching vibrations. Peaks at 1386 cm^−1^ are associated with S=O stretching vibrations of sulfates. Peaks at 1110 cm^−1^ correspond to C–N stretching vibrations of amines, and peaks at 625 cm^−1^ are related to halogenated compounds [19].

After physical characterization, biological characterizations were performed, beginning with the determination of the antibacterial activity of the nanocomposite. The obtained nanocomposite exhibited bactericidal activity against all tested bacteria, with a minimum bactericidal concentration (MBC) of 1.03 µg/mL for most of the tested isolates (Table 3).

**Figure 6:**
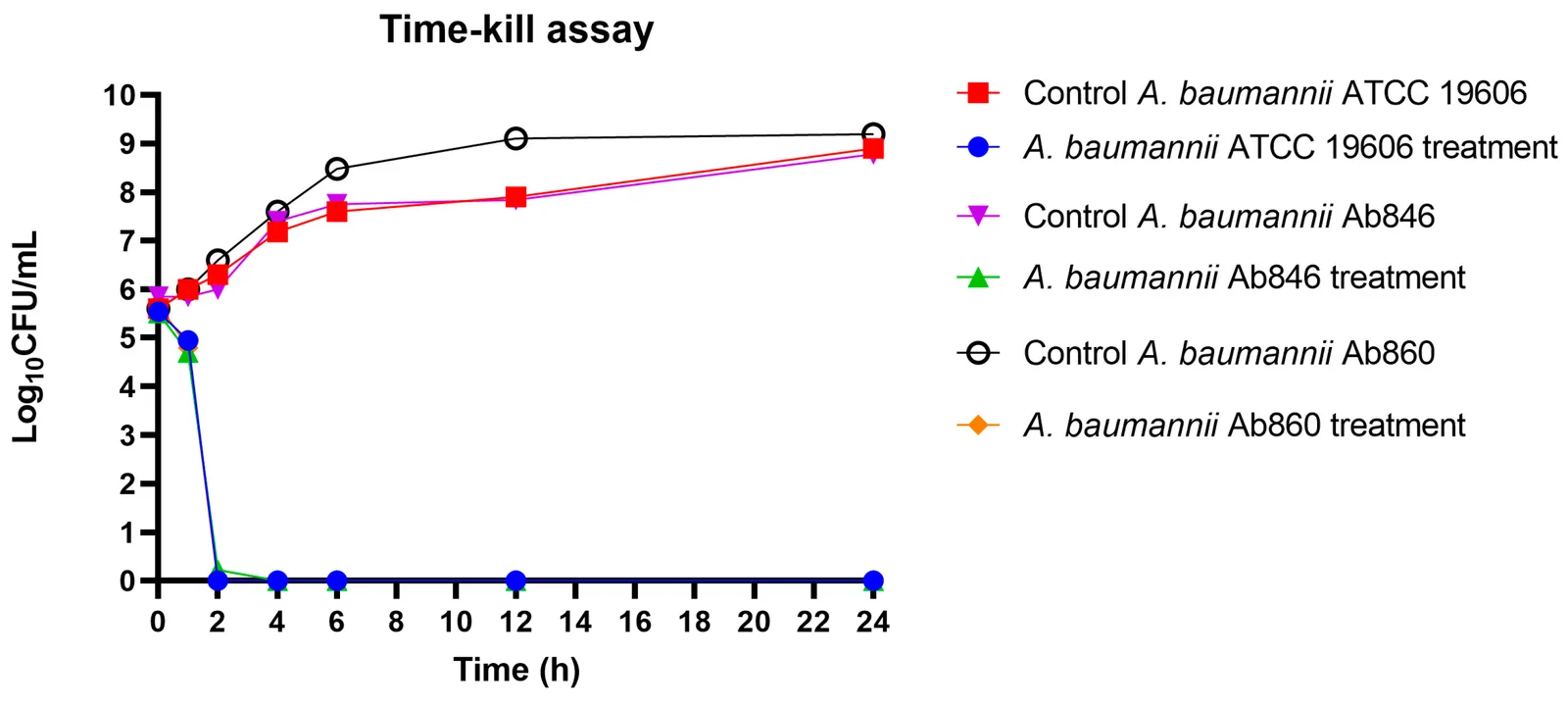
Antibacterial activity kinetics of the silver nanocomposite. A decrease in CFU can be observed starting at 1 h of treatment, with complete reduction from 4 h onward.

Through colony-forming unit (CFU) counting in the time–kill assay, it was possible to observe a reduction in CFU starting at 1 h (Figure 6), with a reduction rate of >95% within 2 h, reaching 99.9% reduction from 4 h onward (Table 4).

The hemolysis rate of human erythrocytes was determined (Supplementary Figure S3). Against human erythrocytes, hemolytic concentrations (>2%) were 25.84 (3.7 ± 1.25%), 51.68 (21.45 ± 7.3%), and 103 µg/mL (54.176 ± 3.7%).

The CC_50_ and SI obtained were 96.79 µg/mL and 93.97, respectively. However, only the lytic activity of the nanocomposite was evaluated here, and further analyses are required to assess its effects on the viability of human cells in order to safely confirm its toxicity.

Selectivity indices above 10 can be considered highly selective and indicative of a favorable therapeutic window [20]. According to the American Society for Testing and Materials, values above 10% are considered hemolytic [21]. Based on this criterion, the concentrations considered hemolytic were those above 25 µg/mL, a value 25 times higher than the MIC. Authors, such as D’Oliveira et al. [22], argue that compounds with SI > 10 are highly selective and present relative in vitro safety [23].

## 4. Discussion

The synthesis of silver nanoparticles using the salt AgNO_3_ is widely documented. The salt dissociates into Ag^+^ and NO_3_^−^ ions. Ag^+^ ions can interact with molecules present in the CFS, being reduced to Ag^0^ or forming AgCl and Ag_3_PO_4_. Subsequently, the silver atoms aggregate into clusters, which grow and stabilize as nanoparticles [23,24].

The antibacterial activity of silver nanoparticles against *A. baumannii* has already been widely demonstrated in the literature [13,25,26]. The MIC values obtained in the present study are comparable to those reported by Allend et al. [25] and Mishra et al. [26], who observed MIC values ranging from 0.460 to 1.95 µg/mL for AgNPs. These remarkably low minimum inhibitory concentration (MIC) and minimum bactericidal concentration (MBC) values may be attributed to the oligodynamic effect of silver, whereby silver retains its antibacterial activity even at very low concentrations [27].

However, despite the extensive literature on the antibacterial activity of AgNPs, relatively few studies have investigated the antimicrobial properties of AgCl nanoparticles and Ag_3_PO_4_ nanoparticles. Tugelbay et al. (2020) demonstrated the antibacterial activity of AgCl/Ag_3_PO_4_ nanoparticles both individually and in combination, suggesting that both AgCl and Ag_3_PO_4_ act as Ag^+^ donors, exerting antibacterial activity [28], although the authors related MIC values of 15- to 250-fold higher against *A. baumannii* in comparison with this work.

While not tested with *A. baumannii*, Aletayeb et al. (2020) observed the antibacterial activity of a AgC/Ag_3_PO_4_ nanocomposite against two strains of *Pseudomonas aeruginosa* [29]. Qureshi et al. (2022) synthesized a Ag/Ag_3_PO_4_ nanocomposite and reported high values of MIC ranging from 125- to 500-fold for *E. coli* [30].

To the best of our knowledge, this is the first study describing the synthesis of a ternary Ag^0^/AgCl/Ag_3_PO_4_ nanocomposite for use as an antimicrobial agent, particularly against *A. baumannii*.

In this study, similar MIC and MBC values were observed between the susceptible strain *A. baumannii* and multidrug-resistant and pan-resistant isolates, indicating that the action of the nanocomposite occurs independently of the resistance phenotype and its mechanisms.

In an *Escherichia coli* model, AgNPs exert their effect mainly through the production of H_2_O_2_, depletion of glutathione levels, and inactivation of glutathione peroxidase, leading to oxidative stress. This stress results in DNA oxidation, causing its fragmentation and inducing an apoptosis-like cell death [31]. The silencing of specific genes in the *E. coli*, such as those involved in RNA biosynthesis, lipopolysaccharide synthesis, and DNA repair proteins, leads to sensitivity to both AgNO_3_ and AgNPs. However, the silencing of genes related to the large ribosomal subunit, peptidoglycan biosynthesis, aminoacyl-tRNA biosynthesis, and DNA biosynthesis results in sensitivity only to AgNO_3_, leading the authors to suggest that AgNPs act more selectively than AgNO_3_ [32]. Furthers works are necessary to understand to evaluate if this phenomenon also occurs in *A. baumannii*.

The action kinetics of the nanocomposite are similar to those of other AgNPs. This can be observed in the study by Deonas et al. [8], who reported that 2 h of exposure was sufficient to reduce 100% of the initial inoculum of *E. coli* ATCC 25922, and in the work of Allend et al. (2022), which showed 100% reduction between 1 and 2 h [13,25]. Compared to the literature, these findings suggest that the nanocomposite exhibits similar action kinetics to nanoparticles composed of a Ag^0^ core.

When compared with the microstrain data, it is likely that the rapid antibacterial activity and low MIC values observed in this study are primarily attributable to the intrinsic mechanism of action of the nanocomposite and the oligodynamic effect of silver rather than to microstrain effects. Smaller crystallites may exhibit higher microstrain, which can increase their specific surface area and consequently enhance ion release, leading to greater generation of reactive oxygen species (ROS) [33].

Regarding erythrocyte toxicity, relatively similar values to those observed in the present study were reported by Choi et al. (2011) for AgNPs containing different stabilizers or after thermal removal of the stabilizing agents [20]. However, these values were lower than those reported by Chen et al., who observed that, within 2 h, nanoparticles ranging from 15 to 100 nm caused up to 20% hemolysis at a concentration of 5 µg/mL [21]. A higher hemolytic rate is more evident in 15 nm nanoparticles; however, as observed in these results, the 26.47 ± 10.9 nm nanocomposite was not toxic at the same tested concentrations.

## 5. Conclusions

Based on the obtained results, it was possible to synthesize nearly spherical silver nanoparticles associated with silver chloride and silver phosphate (Ag/AgCl/Ag_3_PO_4_-NPs), with sizes below 100 nm. Regarding antimicrobial activity, this nanocomposite demonstrated excellent efficacy and rapid kinetics of action against multidrug-resistant and pan-resistant isolates of *A. baumannii*, standing out as a potential therapeutic agent for the control of this pathogen. In addition, the material may be suitable for incorporation into medical devices and equipment, such as catheters, where its antimicrobial properties could help prevent bacterial colonization and biofilm formation, potentially extending the functional lifespan and safety of these devices. Additionally, the data indicate low hemolytic toxicity, suggesting a favorable safety profile at concentrations exceeding the bacteriostatic and bactericidal levels observed in this study. However, these findings should be interpreted with caution, as further investigations are required to comprehensively assess the toxicological profile of the nanocomposite. In particular, in vivo studies are necessary to evaluate potential silver bioaccumulation, tissue distribution, long-term toxicity, and overall biocompatibility before any clinical application can be considered. Taken together, these findings reinforce the promising role of nanotechnology in combating multidrug-resistant bacteria and in the development of highly active antimicrobial agents with reduced toxicity.

## Figures and Tables

**Table 1:** Strains and isolates used in this study.

Strain or Isolate	Site	Year of Isolation	Phenotype
*A. baumannii* ATCC 19606	Reference strain	-	Sensible
*A. baumannii* Ab183	Blood	2020	CRAB, XDR and PolR
*A. baumannii* Ab289	Blood	2023	CRAB, XDR and PolR
*A. baumannii* Ab565	Blood	2022	CRAB and XDR
*A. baumannii* Ab576	Urine	2022	CRAB and XDR
*A. baumannii* Ab791	Blood	2022	CRAB and XDR
*A. baumannii* Ab846	Blood	2021	PDR
*A. baumannii* Ab860	Blood	2023	CRAB, XDR and PolR

CRAB—carbapenem-resistant *A. baumannii.* PDR—pan-drug-resistant. PolR—polymyxin-resistant. XDR—extensively drug-resistant.

**Table 2:** Analytical models for microstructural estimation by XRD.

Phase	JCPDS	Scherrer	Williamson–Hall		SSP	
		Crystallite Size (nm)	Crystallite Size (nm)	Microstrain (%)	Crystallite Size (nm)	Microstrain (%)
Ag	01-089-3722	10,146	8181	−0.0026	11,463	0.0026
AgCl	01-085-1355	12,052	14,373	0.0019	12,668	0.0025
Ag_3_(PO_4_)	01-074-1876	10,101	11,506	0.0020	10,387	0.0031

**Table 3:** Antibacterial activity of the silver nanocomposite against the tested isolates.

Strain or Isolate	MIC (µg/mL)	MBC (µg/mL)	MBC:MIC Ratio	Classification
*A. baumannii* ATCC 19606	1.03	1.03	1	Bactericidal
*A. baumannii* Ab183	1.03	1.03	1	Bactericidal
*A. baumannii* Ab289	1.03	2.06	2	Bactericidal
*A. baumannii* Ab565	1.03	2.06	2	Bactericidal
*A. baumannii* Ab576	1.03	1.03	1	Bactericidal
*A. baumannii* Ab791	1.03	1.03	1	Bactericidal
*A. baumannii* Ab846	1.03	1.03	1	Bactericidal
*A. baumannii* Ab860	1.03	1.03	1	Bactericidal

**Table 4:** Area under the curve (AUC) of the treatments and the control, along with their respective reduction rates. The AUC was used to support the time–kill kinetics data, corroborating the antibacterial activity of the silver nanocomposite. The difference between the treatment AUC and the control AUC indicates that the treatment was effective in reducing growth, as evidenced by the growth reduction rate.

Strain or Isolate	Treatment AUC	Control AUC	Reduction Rate
*A. baumannii* ATCC 19606	7720	187.5	95.88%
*A. baumannii* Ab846	7785	186.8	95.83%
*A. baumannii* Ab860	7650	204.9	96.27%

## Data Availability

The original contributions presented in this study are included in the article and Supplementary Material. Further inquiries can be directed to the corresponding author.

## Supplementary Materials

The following supporting information can be downloaded at: https://www.mdpi.com/article/10.3390/microorganisms14081682/s1, Table S1: Phenotypic resistance profile of the used isolates; Table S2: Calibration dataset for Ag^+^ quantification by absorbance measurements; Table S3: Structural parameters obtained by XRD; Figure S1: Calibration curve for Ag^+^ determination by atomic absorption spectroscopy (AAS); Figure S2: Histogram of particles quantified by transmission electron microscopy (TEM); Figure S3: Calibration curve for hemolytic activity determination by hemoglobin release assay; Document S1: Approval opinion issued by the Research Ethics Committee (REC).
