# Supplementary material for: A Novel Ag/AgCl/Ag3PO4 Nanocomposite Demonstrates Potent Antibacterial Activity Against Multidrug-Resistant and Pan-Resistant Pathogenic Isolates of Acinetobacter baumannii
Source: Microorganisms. 2026 Jul 31;14(8):1682. doi: 10.3390/microorganisms14081682 (PMC13515147; doi:10.3390/microorganisms14081682)
Supplement: Supplementary file 1 [file microorganisms-14-01682-s001.zip › microorganisms-4416265-supplementary.pdf]

Supplementary Information

A novel $Ag/AgCl/Ag_3PO_4$ nanocomposite demonstrates potent antibacterial activity against multidrug-resistant and pan-resistant pathogenic isolates of *Acinetobacter baumannii*

Victor Hugo Montini[1], Laura Santana Buso[1], Anastácia Nikolaos Deonas[1], Gabriel Henrique Maximino Santos[1], Bruna Carolina Gonçalves[1], Maria Luiza Francisconi Lubanco Thomé[1], Paulo Rogério Catarini da Silva[2], Diego Prudencio dos Santos[3], Cesar Ricardo Teixeira Tarley[3], Admilton Gonçalves Oliveira Junior[4], Danielle Lazarin Bidóia[5], Renata Katsuko Takayama Kobayashi[1], Gerson Nakazato[1]*

[1] Laboratory of Basic and Applied Bacteriology, Department of Microbiology, State University of Londrina, Londrina, Brazil.

[2] Laboratory of X-ray Analysis, Multiuser Research Laboratory Center, State University of Londrina, Londrina, Brazil

[3] Department of Chemistry, State University of Londrina, Londrina, Brazil.

[4] Laboratory of Microbial Biotechnology, State Univeristy of Londrina, Londrina, PR, Brazil

5 Department of Clinical Analysis and Biomedicine, State University of Maringá, Maringá, Brazil

Table S1: Phenotypic resistance profile of the used isolates

Isolates	Antimicrobial resistance profile																
	Penicilin		Cephalosporin				Carbapenem		Aminoglycoside			Tetracycline		Fluoroquinolone		Sulfonamide	Peptide	
	mpicilin-sulbacta	eraciclin-Tazobac	Ceftazidime	Cefepime	Cefotaxime	Ceftriaxone	Imipenem	Meropenem	Amikacin	Gentamicin	Tobramycin	Doxycycline	Tetracycline	Ciprofloxacin	Levofloxacin	aoprim/sulfameth	Polymyxin	Colistin
Ab183	12mm	10mm	0mm	0mm	0mm	0mm	0mm	0mm	0mm	0mm	0mm	0mm	0mm	0mm	15mm	0mm	16 µg/mL	32 µg/mL
Ab289	12mm	11mm	9.5mm	9mm	9mm	9mm	9mm	0mm	0mm	0mm	0mm	8.5mm	0mm	0mm	15.5mm	0mm	4 µg/mL	8 µg/mL
Ab565	8mm	8mm	0mm	0mm	0mm	0mm	0mm	0mm	9mm	14mm	15mm	13mm	13mm	0mm	0mm	10mm	1 µg/mL	0.5 µg/mL
Ab576	9mm	0mm	0mm	10mm	0mm	0mm	0mm	0mm	0mm	0mm	0mm	0mm	0mm	0mm	0mm	0mm	1 µg/mL	2 µg/mL
Ab791	8mm	8mm	0mm	0mm	0mm	0mm	0mm	0mm	0mm	0mm	0mm	0mm	0mm	0mm	15.5mm	0mm	16 µg/mL	32 µg/mL
Ab846	0mm	0mm	0mm	0mm	0mm	0mm	0mm	0mm	0mm	0mm	0mm	0mm	0mm	0mm	13mm	0mm	16 µg/mL	32 µg/mL
Ab860	8mm	0mm	0mm	0mm	0mm	0mm	0mm	0mm	0mm	0mm	0mm	0mm	0mm	0mm	14.5mm	0mm	16 µg/mL	32 µg/mL

Legend
Sensible
Intermediate
Resistant

Table S2- Calibration dataset for Ag^+ quantification by absorbance measurements

**Concentration Ag^+ (mg L^{-1})**	**Abs 1**	**Abs 2**	**Abs 3**	**Mean**	**Deviation**	**RSD**
**1**	0,123	0,125	0,126	0,124667	0,001528	1,225288
**2**	0,212	0,215	0,213	0,213333	0,001528	0,716027
**3**	0,312	0,309	0,305	0,308667	0,003512	1,13776
**4**	0,391	0,392	0,395	0,392667	0,002082	0,530136
**5**	0,48	0,482	0,484	0,482	0,002	0,414938
**6**	0,556	0,558	0,553	0,555667	0,002517	0,452899
**7**	0,614	0,613	0,63	0,619	0,009539	1,541097
**8**	0,673	0,668	0,683	0,674667	0,007638	1,132059
**9**	0,755	0,756	0,763	0,758	0,004359	0,575053
**10**	0,816	0,82	0,834	0,823333	0,009452	1,147971
**Blank**	0	0	0	0	0	#DIV/0!
**Ag Nanocomposite**	0,136	0,135	0,137	0,136	0,001	0,735294118

Table S3- Structural parameters obtained by XRD

No.	Position (2θ)	h	k	l	d (Å)	FWHM (°2θ)	Phase
1	20,9133	0	1	1	4,24428	0,01476	Ag3 (PO4)_27843
2	20,9658	0	1	1	4,24429	0,014764	Ag3 (PO4)_27843
3	27,8708	1	1	1	3,19854	0,012573	Ag Cl_64734
4	27,9414	1	1	1	3,19855	0,012579	Ag Cl_64734
5	29,7365	0	0	2	3,00198	0,015362	Ag3 (PO4)_27843
6	29,812	0	0	2	3,00199	0,015369	Ag3 (PO4)_27843

7	32,2886	0	0	2	2,77029	0,01289<br>1	Ag<br>Cl_6473<br>4
8	32,371	0	0	2	2,77029	0,01289<br>6	Ag<br>Cl_6473<br>4
9	33,3405	0	1	2	2,68525	0,01567<br>1	Ag3 (P<br>O4)_278<br>43
10	33,4258	0	1	2	2,68525	0,01567<br>8	Ag3 (P<br>O4)_278<br>43
11	36,6282	1	1	2	2,45142	0,01598<br>4	Ag3 (P<br>O4)_278<br>43
12	36,7224	1	1	2	2,45142	0,01599<br>4	Ag3 (P<br>O4)_278<br>43
13	38,1526	1	1	1	2,3569	0,01524<br>2	Ag_443<br>87
14	38,2511	1	1	1	2,3569	0,01524	Ag_443<br>87
15	42,5456	0	2	2	2,12315	0,01662<br>8	Ag3 (P<br>O4)_278<br>43
16	42,6565	0	2	2	2,12315	0,01664	Ag3 (P<br>O4)_278<br>43
17	44,3404	0	0	2	2,04129	0,01513<br>2	Ag_443<br>87
18	44,4564	0	0	2	2,04129	0,01513	Ag_443<br>87
19	46,3023	0	2	2	1,95926	0,01422<br>1	Ag<br>Cl_6473<br>4

20	46,424	0	2	2	1,95926	0,01423 3	Ag Cl_6473 4
21	47,8589	0	1	3	1,89911	0,01729 4	Ag3 (P O4)_278 43
22	47,9852	0	1	3	1,89911	0,01731 2	Ag3 (P O4)_278 43
23	52,758	2	2	2	1,73371	0,01798 9	Ag3 (P O4)_278 43
24	52,8991	2	2	2	1,73371	0,01801	Ag3 (P O4)_278 43
25	54,9014	1	1	3	1,67098	0,01529 4	Ag Cl_6473 4
26	55,0493	1	1	3	1,67098	0,01531 4	Ag Cl_6473 4
27	55,0894	0	2	3	1,66572	0,01834 7	Ag3 (P O4)_278 43
28	55,2379	0	2	3	1,66573	0,01837 1	Ag3 (P O4)_278 43
29	57,3564	1	2	3	1,60516	0,01871 3	Ag3 (P O4)_278 43
30	57,5122	1	2	3	1,60516	0,01874	Ag3 (P O4)_278 43
31	57,5636	2	2	2	1,59987	0,01567	Ag Cl_6473 4

32	57,72	2	2	2	1,59988	0,015692	AgCl_64734
33	61,7291	0	0	4	1,50153	0,019473	Ag3 (PO4)_27843
34	61,8992	0	0	4	1,50153	0,019504	Ag3 (PO4)_27843
35	63,8478	0	1	4	1,45671	0,019865	Ag3 (PO4)_27843
36	64,0252	0	1	4	1,45672	0,0199	Ag3 (PO4)_27843
37	64,4961	0	2	2	1,44363	0,014759	Ag_44387
38	64,6758	0	2	2	1,44363	0,014755	Ag_44387
39	65,9287	1	1	4	1,41569	0,02027	Ag3 (PO4)_27843
40	65,9287	0	3	3	1,41569	0,02027	Ag3 (PO4)_27843
41	66,1133	1	1	4	1,41569	0,020307	Ag3 (PO4)_27843
42	66,1133	0	3	3	1,41569	0,020307	Ag3 (PO4)_27843
43	67,5492	0	0	4	1,38561	0,017277	AgCl_64734
44	67,7397	0	0	4	1,38561	0,01731	AgCl_64734

45	69,9946	0	2	4	1,34307	0,02111	Ag3 (PO4)_27843
46	70,194	0	2	4	1,34307	0,021153	Ag3 (PO4)_27843
47	71,9874	1	2	4	1,31071	0,02155	Ag3 (PO4)_27843
48	72,1943	1	2	4	1,31071	0,021595	Ag3 (PO4)_27843
49	73,9578	2	3	3	1,28059	0,022	Ag3 (PO4)_27843
50	74,1723	2	3	3	1,28059	0,02205	Ag3 (PO4)_27843
51	74,5712	1	3	3	1,27156	0,018621	Ag Cl_64734
52	74,7881	1	3	3	1,27157	0,018665	Ag Cl_64734
53	76,8544	0	2	4	1,23938	0,019101	Ag Cl_64734
54	77,0804	0	2	4	1,23938	0,01915	Ag Cl_64734
55	77,4595	1	1	3	1,2312	0,014505	Ag_44387
56	77,6879	1	1	3	1,2312	0,0145	Ag_44387
57	77,8435	2	2	4	1,22609	0,022948	Ag3 (PO4)_27843

58	78,0735	2	2	4	1,22609	0,023005	Ag3 (PO4)_27843
59	79,764	0	3	4	1,20132	0,023445	Ag3 (PO4)_27843

Figure S1. Calibration curve for Ag^+ determination by atomic absorption spectroscopy (AAS)

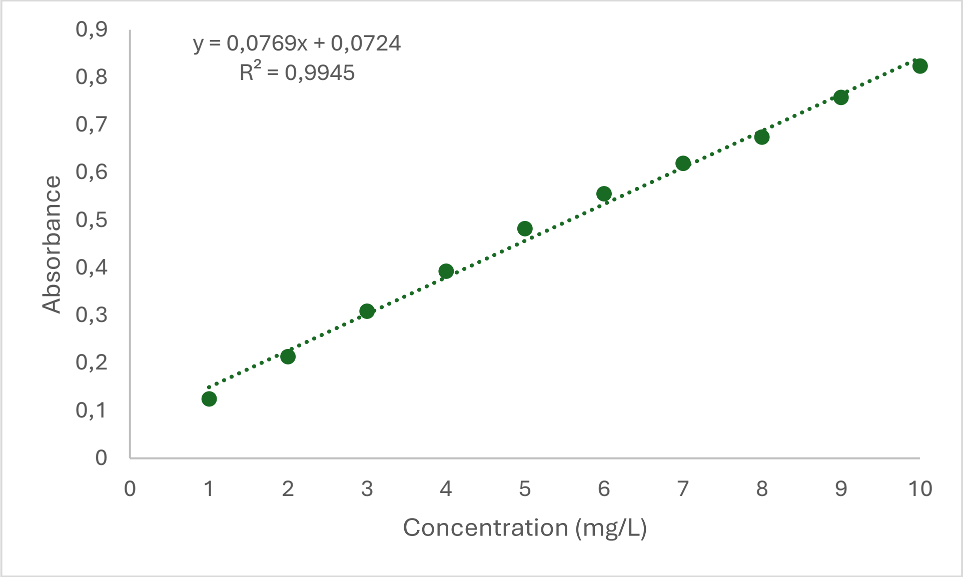

Figure S2. Histogram of particles quantified by transmission electron microscopy (TEM).

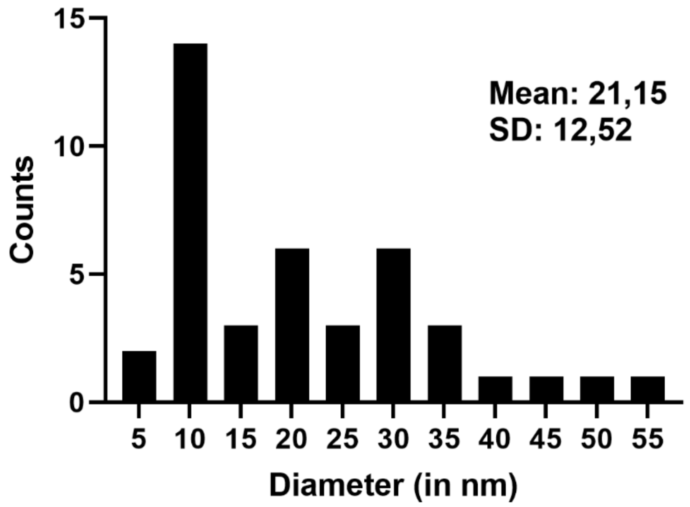

Figure S3. Calibration curve for hemolytic activity+ determination by hemoglobina release assay.

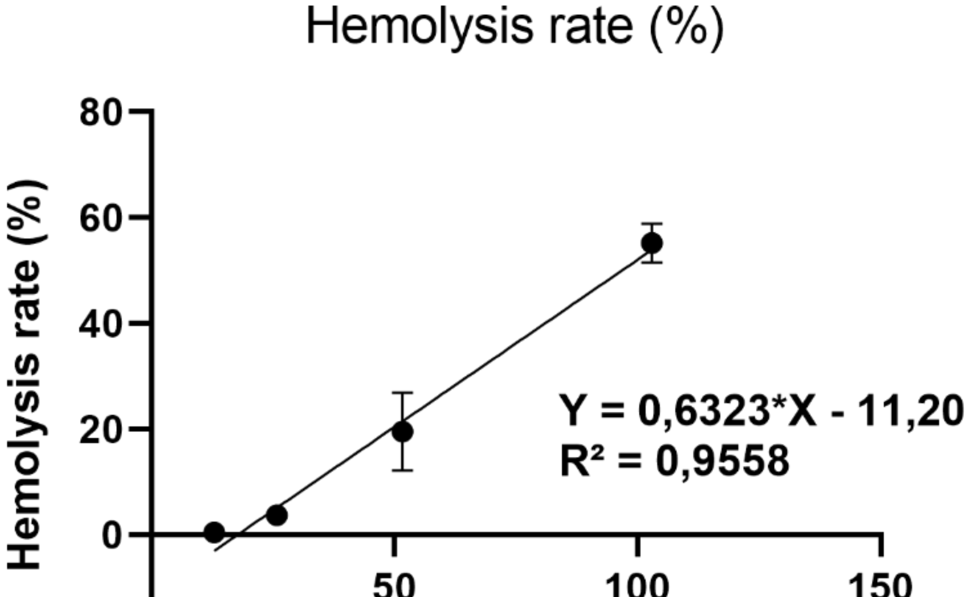

Document S1: Approval opinion issued by the Research Ethics Committee (REC)

PARECER CONSUBSTANCIADO DO CEP

DADOS DO PROJETO DE PESQUISA

Título da Pesquisa: AVALIAÇÃO DA TOXICIDADE DE COMPOSTOS ALTERNATIVOS COM AÇÃO ANTIMICROBIANA

Pesquisador: Renata Katsuko Takayama Kobayashi

Área Temática:

Versão: 3

CAAE: 47661115.0.0000.5231

Instituição Proponente: CCB - Departamento de Microbiologia

Patrocinador Principal: Financiamento Próprio

DADOS DO PARECER

Número do Parecer: 1.268.019

Apresentação do Projeto:

Trata-se de um projeto para avaliar a toxicidade de compostos alternativos com ação antimicrobiana, pois é sabido que a disseminação de microrganismos resistentes aos antimicrobianos convencionais é um problema que está afetando o recinto hospitalar, o ambiente e a comunidade de forma geral.

Objetivo da Pesquisa:

Objetivo Primário:

Avaliar ação citotóxica de compostos antimicrobianos.

Objetivo Secundário:Determinar a concentração tóxica em hemácias (CC50; ou seja, concentração que danifica 50% das células)de compostos antimicrobianos alternativos. Comparar os resultados encontrados nessa linhagem celular

com os resultados encontrados em células tumorais (HEp-2, HeLa, LLCMK2).

Avaliação dos Riscos e Benefícios:

Riscos: O risco será mínimo. O risco é próprio de uma coleta de sangue, como dor, hematoma ou outro desconforto no local da coleta. Raramente desmaio ou infecções no local de punção podem ocorrer. Por isso, o procedimento será realizado por profissionais qualificados e normas de biossegurança serão utilizadas, como uso de luvas, jaleco e técnicas assépticas. Além disso, todos

Endereço: LABESC - Sala 14
Bairro: Campus Universitário **CEP:** 86.057-970
UF: PR **Município:** LONDRINA
Telefone: (43)3371-5455 **E-mail:** cep268@uel.br

os materiais que entrarem em contato com o sangue, seja por meio da coleta ou do experimento, serão descartados adequadamente.

Em caso de desconforto ou outros danos devido ao procedimento de coleta de sangue, o voluntário será encaminhado para a Divisão de Assistência à Saúde da Comunidade (DASC – UEL).

Benefícios:

Esse estudo irá auxiliar no processo de desenvolvimento de novos agentes antimicrobianos. Após testes de toxicidade, tais agentes podem ganhar potencial para serem utilizados em indústrias farmacêuticas, alimentícias, hospitais, clínicas, entre outros.

Comentários e Considerações sobre a Pesquisa:

Trata-se de um estudo importante para área pois, a disseminação de microrganismos resistentes aos antimicrobianos convencionais é um problema que está afetando o recinto hospitalar, o ambiente e a comunidade de forma geral. A pesquisadora pertence a um grupo de pesquisa que vem avaliando a resistência de microrganismos isolados de humanos e alimentos a antibióticos comerciais, principalmente no aspecto epidemiológico. O conhecimento resultante desse estudo poderá reverter em benefícios no desenvolvimento de novos agentes para controle de infecções bacterianas, visando métodos alternativos para o combate às bactérias, principalmente às multirresistentes aos antimicrobianos.

Considerações sobre os Termos de apresentação obrigatória:

Os termos de apresentação obrigatória estão corretos.

Recomendações:

Conclusões ou Pendências e Lista de Inadequações:

O pesquisador atendeu satisfatóriamente as pendências levantadas pelo CEP, apresentando o documento da DASC, com ciencia da pesquisa e se compromentendo em atender os participante do estudo caso, for necessário. E adequou o cronograma. Projeto está aprovado

Considerações Finais a critério do CEP:

Prezado (a) Pesquisador (a),

Este é seu parecer final de aprovação, vinculado ao Comitê de Ética em Pesquisas Envolvendo Seres Humanos da Universidade Estadual de Londrina. É sua responsabilidade imprimi-lo para apresentação aos órgãos e/ou instituições pertinentes.

Coordenação CEP/UEL.

Este parecer foi elaborado baseado nos documentos abaixo relacionados:

Tipo Documento	Arquivo	Postagem	Autor	Situação
Informações Básicas do Projeto	PB_INFORMAÇÕES_BÁSICAS_DO_PROJETO_549298.pdf	18/09/2015 21:42:46		Aceito
Outros	dasc.pdf	18/09/2015 21:42:13	Renata Katsuko Takayama Kobayashi	Aceito
Projeto Detalhado / Brochura Investigador	Projeto3.pdf	18/09/2015 21:40:00	Renata Katsuko Takayama Kobayashi	Aceito
TCLE / Termos de Assentimento / Justificativa de Ausência	TCLE.pdf	09/09/2015 08:37:39	Renata Katsuko Takayama Kobayashi	Aceito
Folha de Rosto	Doc1.pdf	09/09/2015 08:28:09	Renata Katsuko Takayama Kobayashi	Aceito

Situação do Parecer:
Aprovado

Necessita Apreciação da CONEP:
Não

LONDRINA, 08 de Outubro de 2015

Assinado por:
Alexandrina Aparecida Maciel Cardelli
(Coordenador)
